# Treatment of an accident of imidacloprid poisoning

**DOI:** 10.3389/fphar.2024.1421437

**Published:** 2024-07-24

**Authors:** Mei Zeng, Mengdi Shi, Xiangdong Jian, Laidong Dong

**Affiliations:** ^1^ Department of Poisoning and Occupational Diseases, Emergency Medicine, Qilu Hospital of Shandong University, Cheeloo College of Medicine, Shandong University, Jinan, Shandong, China; ^2^ Department of Occupational and Environmental Health, School of Public Health, Cheeloo College of Medicine, Shandong University, Jinan, Shandong, China; ^3^ Qilu Hospital of Shandong University, Cheeloo College of Medicine, Shandong University, Jinan, Shandong, China

**Keywords:** imidacloprid, acute poisoning, toxicant detection, hemoperfusion, case series study

## Abstract

**Objective:** Accidental oral imidacloprid poisoning occurred in a family in Shandong, China, in May 2023. This study aimed to analyze the clinical characteristics of this imidacloprid poisoning event and investigated the detection of toxicants.

**Methods:** Clinical data of four patients with oral imidacloprid poisoning were collected and retrospectively analyzed. The relevant literature was then reviewed.

**Results:** Four patients from the same family received different oral doses of imidacloprid. The main clinical manifestations were digestive and neurological symptoms, including nausea, vomiting, and varying degrees of consciousness. Laboratory tests showed an increased white blood cell count, neutrophil proportion, and mild elevation of transaminase and urea nitrogen levels in some patients. Following comprehensive treatment, which included hemoperfusion, gastric lavage, total gastrointestinal decontamination, and drug symptomatic treatment, the patient’s symptoms were quickly relieved, and the concentration of imidacloprid in the blood rapidly decreased.

**Conclusion:** Toxicant detection is an important criterion for the differential diagnosis of poisoning and is helpful for disease assessment, treatment plan formulation, and in determining patient prognosis.

## 1 Introduction

Imidacloprid is one of the most widely used neonicotinoid insecticides worldwide (Abd-Elhakim et al., 2023). It is chemically similar to nicotine and mainly acts on nicotinic acetylcholine receptors (nAChRs) (Naveen et al., 2022). Imidacloprid has long been considered safer than other insecticides because of its favorable toxicological profile (Naveen et al., 2022); however, there has been an increase in the incidence of poisoning, resulting in serious damage to organs, including the heart (Huang et al., 2006), kidneys, and liver (Sriapha et al., 2020a), and even leading to death (Iyyadurai et al., 2010; Naveen et al., 2022). Mice toxicity studies have also reported hepatotoxicity and nephrotoxicity histologically and biochemically (Arfat et al., 2014; Perananthan et al., 2021). Moreover, it has been reported that imidacloprid may cause rare clinical manifestations such as methemoglobinemia (Chadalavada and Baddam, 2023). This has raised concerns about imidacloprid poisoning. In the reported cases, the medical history, circumstantial evidence, and clinical features have formed the basis for the clinical diagnosis of imidacloprid poisoning; a diagnosis using laboratory tests is not universal. To date, there is little information on imidacloprid toxicity in humans and more data are needed.

In May 2023, we admitted four patients from the same family with accidental oral imidacloprid poisoning. All four patients underwent multiple blood toxicant tests, were cured, and were discharged after comprehensive treatment. Here, we report these cases in detail and review the relevant literature.

## 2 Materials and methods

The clinical data of four patients with oral imidacloprid poisoning were collected from electronic medical records and retrospectively analyzed. This study was approved by the Ethics Committee of the Qilu Hospital of Shandong University (Jinan, Shandong Province) (Ethics No. KYLL-202106(KS)-040). Written informed consent for the publication of this study was obtained from all patients.

### 2.1 General patient information

The four patients were from the same family and aged between 33 and 55 years. After regaining consciousness, a detailed medical history was obtained. The patients had been healthy and had no history of diseases or allergies. At noon on May 16, the patients received an unspecified dose of imidacloprid orally. Four patients shared a bottle of imidacloprid (emulsion, 300 g/bottle, 5% active ingredient content), with case 4 taking the smallest amount. Approximately 30 min later, they were transported to the poisoning department of our hospital by ambulance and immediately treated with gastric lavage.

### 2.2 Clinical characteristics and treatment

The four patients developed nausea and vomiting soon after insecticide intake; the vomit contained food residue and pesticide. Soon after, they developed varying degrees of disturbances in consciousness, such as lethargy and light coma. In addition, all four patients had elevated blood pressure levels. The primary symptoms and signs are listed in Table 1. Laboratory tests revealed an elevated white blood cell count, neutrophil ratio, and the levels of some inflammatory factors. Three patients had mild elevations in transaminase levels (case 1, 2, and 4), and three patients had transient mild elevations in urea nitrogen (case 1, 2, and 3). The initial concentration of imidacloprid was 3.423 μg/mL, 4.935 μg/mL, 2.624 μg/mL, and 0.013 μg/mL (reference value < 0.001 μg/mL). None of the patients had abnormalities in troponin I, cholinesterase, or coagulation marker levels. The main test results are presented in Tables 2 and 3. Chest computed tomography imaging results of the four patients are shown in Table 1, which mainly showed exudative or hypostatic changes, pleural thickening, a small amount of pleural effusion, and fibrous foci.

**TABLE 1:** Main clinical manifestations and imaging examination results of all patients.

Items	Sex	Age	Body weight (kg)	Main clinical manifestation	GCS	Vital signs	When did their clinical signs stabilize	Chest CT findings
**Case 1**	Female	53	67	Nausea, emesis, light coma	7	PR: 89 b/m RR: 13 b/m BP: 188/105 mmHg	3 days after poisoning	Patchy ground-glass opacities in the lower lobes of both lungs, bilateral pleural thickening, and a small amount of bilateral pleural effusion
**Case 2**	Male	55	76	Nausea, emesis, drowsiness	14	PR: 96 b/m RR: 17 b/m BP: 184/126 mmHg	3 days after poisoning	Few interstitial changes in the right lung; Bilateral bronchitis, bilateral pulmonary fibrosis foci, and bilateral pleural thickening
**Case 3**	Female	33	62	Nausea, emesis, light coma	7	PR: 87 b/m RR: 11 b/m BP: 162/102 mmHg	3 days after poisoning	Mild hypostatic changes in both lungs, bilateral pulmonary fibrosis foci, and a small bilateral pleural effusion
**Case 4**	Female	33	57	Nausea, emesis, drowsiness, tachypnea	14	PR: 98 b/m RR: 26 b/m BP: 137/93 mmHg	2 days after poisoning	Mild hypostatic changes in both lungs

GCS, glasgow coma scale; PR, pulse rate; RR, respiratory rate; BP, blood pressure; b/m, beats/minute.

**TABLE 2:** Main laboratory workup results of the patients at different time-points following hospital admission.

Items	Time	Case 1	Case 2	Case 3	Case 4
**WBC (3.5**–**9.5** × **10** ^**9**^ **/L**)	Day 1	7.5	**9.9**	**10.6**	9.1
	Day 3	**12.5**	**13.5**	**9.6**	**11.7**
	Day 7	**11.6**	**15.4**	**11.5**	**12.9**
	Day 14	8.7	5.8	4.3	9.4
**NEU% (40.0**–**75.0%)**	Day 1	**79.1**	**83.4**	**82.3**	**84.3**
	Day 3	**75.5**	**75.8**	73.2	73.3
	Day 7	**79.3**	**80.4**	72.4	73.2
	Day 14	74.8	53.7	51.9	63.7
**HGB (115.0**–**150.0** ** ** **g/L)**	Day 1	119.0	139.0	**101.0**	117.0
**ALT (0.0**–**35.0 IU/L)**	Day 1	**85.0**	33.0	23.0	16.0
	Day 3	**44.0**	21.0	15.0	7.0
	Day 7	**125.0**	**270.0**	26.0	**47.0**
	Day 14	**52.0**	**88.0**	23.0	19.0
**AST (14.0**–**36.0 IU/L)**	Day 1	**68.0**	31.0	24.0	23.0
	Day 3	22.0	20.0	13.0	11.0
	Day 7	**52.0**	**237.0**	16.0	25.0
	Day 14	**38.0**	26.0	16.0	17.0
**TBIL (3.0**–**22.0** ** ** **μmol/L)**	Day 1	6.0	14.0	13.0	7.0
**BUN (2.5**–**6.1** ** ** **mmol/L)**	Day 1	**7.1**	**8.1**	3.7	3.3
	Day 3	5.1	**7.7**	4.9	3.8
	Day 7	**7.3**	**10.1**	**8.5**	3.1
	Day 14	4.5	5.8	3.5	3.1
**Cr (46.0**–**106.0** ** ** **μmol/L)**	Day 1	59.0	73.0	49.0	50.0
**CTNI (<17.5** ** ** **ng/L)**	Day 1	2.6	3.3	1.7	2.7
**CHE (4650.0**–**10,440.0 IU/L)**	Day 1	9537.0	7541.0	7484.0	7663.0
**PT (8.8**–**13.8** **s)**	Day 1	11.8	13.1	13.3	12.7
**APTT (26.0**–**42.0)**	Day 1	35.1	33.7	35.3	33.6

Day 1, Day of poisoning. WBC, white blood cell; NEU, neutrophils; HGB, hemoglobin; ALT, alanine aminotransferase; AST, aspartate aminotransferase; TBIL, total bilirubin; BUN, blood urea nitrogen; Cr, serum creatinine; CTNI, cardiac troponin I; CHE, cholinesterase; PT, prothrombin time; APTT, activated partial thromboplastin time. Abnormal test results are indicated in bold.

**TABLE 3:** Inflammatory factor levels at admission.

Items	Case 1	Case 2	Case 3	Case 4
**IL-6 (0**–**7** ** ** **pg/mL)**	2.32	<2.00	2.21	**11.40**
**IL-1b** **(0**–**5** ** ** **pg/mL)**	**>1000.00**	<5.00	<5.00	**7.24**
**IL-2R** **(223**–**710** ** ** **u/mL)**	350.00	369.00	248.00	**156.00**
**IL-8** **(0**–**62** ** ** **pg/mL)**	5.53	5.78	<5.00	<5.00
**IL-10** **(0**–**9.1** ** ** **pg/mL)**	<5.00	<5.00	<5.00	<5.00
**TNF** **(0**–**8.1** ** ** **pg/mL)**	**15.20**	5.81	4.51	<4.00

IL-6, interleukin 6; IL-1b, interleukin-1B; IL-2R, interleukin-2 receptor; IL-8, interleukin-8; IL-10, interleukin-10; TNF, tumor necrosis factor alpha. Abnormal test results are indicated in bold.

**FIGURE 1:**
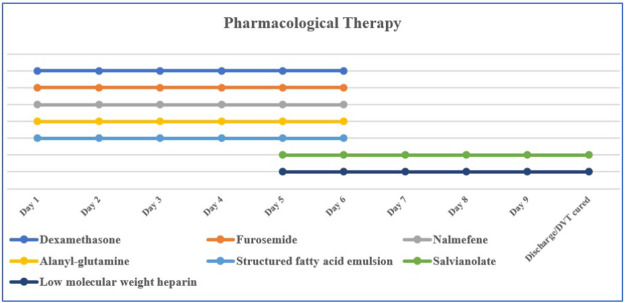
Daily pharmacological therapy. DVT**,** deep venous thrombosis.

Currently, there is no specific antidote for imidacloprid poisoning; comprehensive treatment is the main treatment. Commonly used therapeutic drugs are dexamethasone (10 mg/day, intravenous drip), furosemide (20 mg, twice per day, intravenous injection), nalmefene (0.1 mg, twice per day, intravenous injection), alanyl-glutamine (20 g/day, intravenous drip), salvianolate (200 g/day, intravenous drip), structured fatty acid emulsion (50 g/day, intravenous drip), and low molecular weight heparin (5,000 IU/day, hypodermic injection) (Figure 1). Total gastrointestinal decontamination was performed after gastric lavage. The specific method was as follows: montmorillonite powder (30 g) was dissolved in 250 mL of 20% mannitol and administered in divided doses; activated charcoal (powder) (30 g) was dissolved in 20% mannitol (250 mL) and administered in divided doses. The first dose was administered within 2 h after admission, followed by divided doses on days 2–4. HA330 hemoperfusion was simultaneously administered; on the first day, hemoperfusion was performed twice at an interval of 4 h, and once a day on days two and three. Each hemoperfusion procedure lasted 2 h.

## 3 Results

**FIGURE 2:**
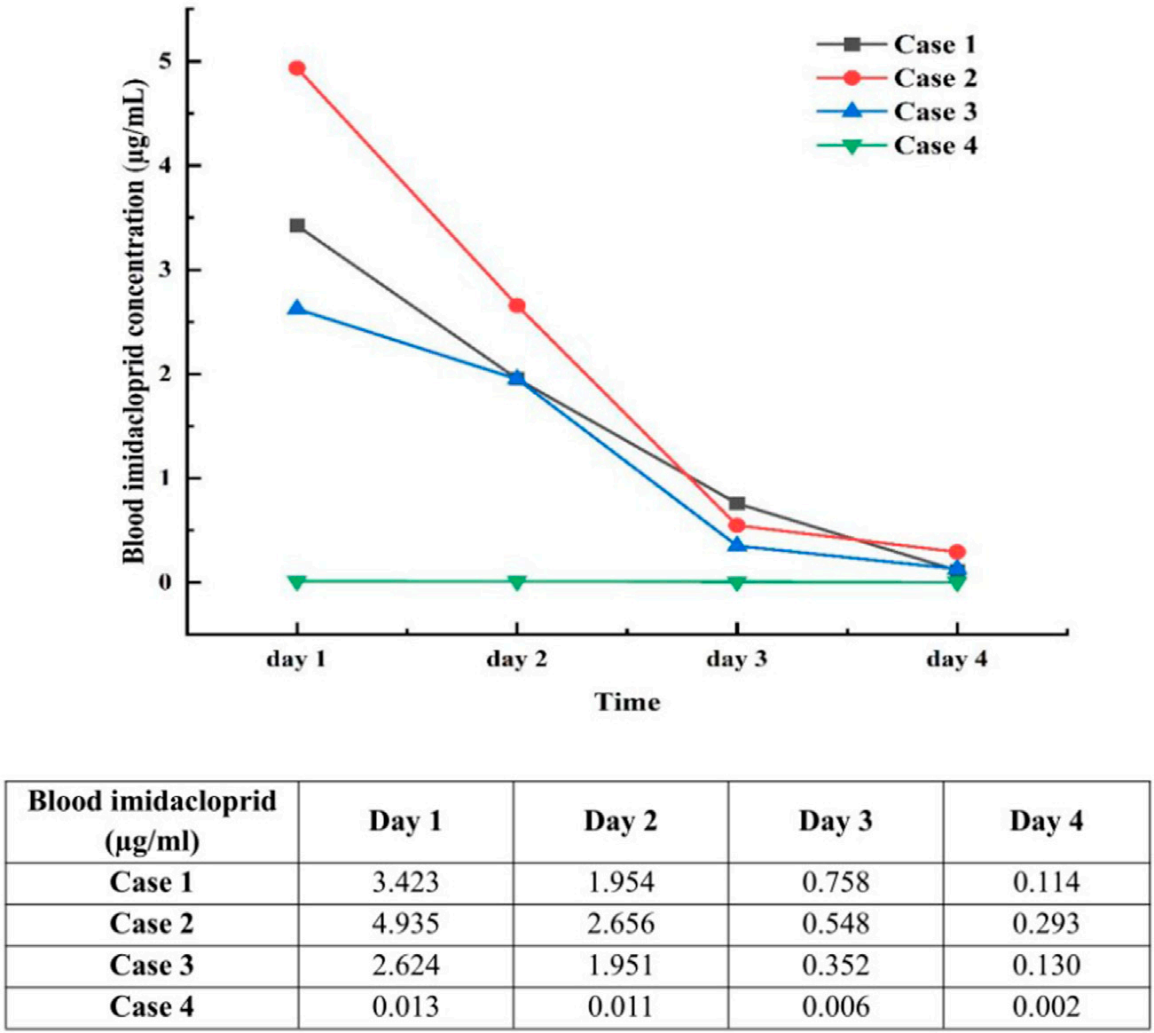
Daily changes in blood imidacloprid concentrations for the first 4 days after poisoning. Day 1, Day of poisoning; reference range: <0.001 μg/mL.

The symptoms gradually improved in all patients, with a rapid decrease in the blood imidacloprid concentration (Figure 2). Patients one and four were discharged after 10 days of treatment, while patients two and three were cured and discharged after 14 days.

## 4 Discussion

Imidacloprid is the world’s largest selling insecticide, which mainly acts on nAChRs. Imidacloprid initially stimulates nAChRs and subsequently inhibits them (Shadnia and Moghaddam, 2008; Karatas, 2009; Naveen et al., 2022). In humans, it can be absorbed through ingestion, skin, or inhalation, with oral ingestion being more severe than the other routes (Bhatta et al., 2023). Imidacloprid is generally considered to be relatively safer than other insecticides, such as organophosphorus, carbamates, and organochlorines (Naveen et al., 2022). Firstly, the binding affinity of imidacloprid to insect nicotine receptors is much higher than that in vertebrates. Secondly, imidacloprid is highly water-soluble, which effectively reduces its ability to cross the blood–brain barrier, thereby reducing its toxicity to the central nervous system (Tomizawa and Casida, 2005; Phua et al., 2009). Yet despite this, clinical studies observed neurological symptoms in 17.6% of imidacloprid exposure cases (Perananthan et al., 2021; Naveen et al., 2022). Reported cases of imidacloprid poisoning usually present with mild gastrointestinal and neurological symptoms. Neurological involvement can lead to dizziness, somnolence, disorientation, and a comatose state. The initial stimulation of the autonomic nervous system leads to sweating, cardiomyopathy, tachycardia, hypertension, coronary spasms, and myocardial ischemia, and subsequent inhibition can lead to arrhythmias, hypotension, and bradycardia (Karatas, 2009; Mohamed et al., 2009; Yeh et al., 2010; Lin et al., 2013). Imidacloprid exposure has been suggested to induce lysosomal dysfunction and cell death in human astrocytes and fibroblasts, which may be associated with the neurological symptoms of imidacloprid poisoning (Eriksson et al., 2023). Animal experiments suggest that imidacloprid can induce pyroptosis in Kupffer cells through P2X7 and further induce liver injury (Pei et al., 2023). In addition, it can activate the apoptotic pathway through the excessive production of reactive oxygen species, causing liver and kidney injury (Hassanen et al., 2022). Acute oral exposure to imidacloprid was reported to induce apoptosis and autophagy in the midgut of honeybee workers, which may affect their physiological digestibility (Lenise Silva et al., 2022). The patient reported here also presented with gastrointestinal and nervous system symptoms accompanied by hypertension and a mild transient elevation of transaminase and urea nitrogen. The peaks of liver and kidney tests were delayed, which may be related to the time required for imidacloprid to damage cells. In addition, imidacloprid can cause male reproductive toxicity; its cytotoxic effect on rat LC-540 cells was suggested to be related to mitochondrial damage and the fragmentation of cytoskeletal proteins (Mia et al., 2023).

Despite its safety, severe imidacloprid-induced poisonings are common (Naveen et al., 2022). There is growing evidence that imidacloprid may cause damage to cardiac, renal, and other organs. A series of serious complications have been reported, including neurological sequelae, acute kidney injury due to rhabdomyolysis, ischemic and metabolic encephalopathy, ventricular fibrillation, multiorgan failure, and even death (Huang et al., 2006; Agarwal and Srinivas, 2007; Karatas, 2009; Mohamed et al., 2009; Phua et al., 2009; Yeh et al., 2010; Fuke et al., 2014; Sriapha et al., 2020a; Perananthan et al., 2021; Naveen et al., 2022). In particular, since 2007, new dosage forms of imidacloprid containing unknown solvents have been introduced; thus, the toxicity spectrum has changed, and reported deaths and cases requiring mechanical ventilation have increased (Perananthan et al., 2021). Imidacloprid is usually fatal when ingested with other poisons, such as organophosphorus, carbamate, and alcohol. One retrospective study suggested that most patients with imidacloprid poisoning experience only mild toxicity. Despite the low case fatality rate, only a small number of patients with initially mild symptoms died. Close observation and monitoring should be considered in patients with a large intake or warning signs, such as cardiovascular effects, central nervous system effects, dyspnea, and sweating (Sriapha et al., 2020b).

Information on imidacloprid toxicity in humans is scarce. We were able to gather only a small number of published case reports and studies. There have been no toxicokinetic studies on imidacloprid toxicity in humans. The LD_50_ values are 380–650 mg/kg in rats and 130–170 mg/kg in mice. Imidacloprid absorption is rapid and extensive (95%) in rats after ingestion and is evenly and rapidly distributed in all tissues. After 48 h, the highest residues were found in the liver, kidneys, lungs, and skin. Furthermore, approximately 70%–80% is excreted in urine within 48 h, and 20%–30% is excreted in feces (Naveen et al., 2022).

Most clinicians formulate clinical diagnoses based on medical history, circumstantial evidence, and clinical features, but toxicological detection is rarely supplemented. Currently, no specific diagnostic tools or markers can be used to diagnose imidacloprid poisoning using laboratory tests. Liquid chromatography/mass spectrometry (Proença et al., 2005), gas chromatography/mass spectrometry (Buchweitz et al., 2019), and high-performance liquid chromatography/photodiode array detector (Fuke et al., 2014) can be used to screen and quantify imidacloprid compounds. To confirm the diagnosis and assess the changes in blood imidacloprid concentrations during standard treatment, all patients in this study underwent daily plasma imidacloprid concentration measurements during the first 4 days of intoxication. The concentration of imidacloprid in the blood of cases 1–3 increased significantly after the poisoning and showed an evident continuous downward trend. These three patients still had some level of imidacloprid in their blood 4 days after ingestion. The blood imidacloprid concentration in case 4 was relatively low, which may be related to the small amount of poison ingested by the patient.

There is no specific antidote for imidacloprid poisoning, and treatment is mainly symptomatic and supportive. High doses of imidacloprid can inhibit butyrylcholinesterase, and when a patient has bradycardia and sweating, the doctor may mistake organophosphate poisoning for mixed organophosphates. Previous studies justified the use of atropine in cases of bronchial leakage, airway endangerment, and bradycardia; however, oximes are ineffective in treating neonicotinoid insecticide poisoning as they may increase toxicity by increasing nicotine-related symptoms, such as tachycardia, hypertension, and muscle weakness (Forrester, 2014; Hassanen et al., 2022). Therefore, toxicant testing is particularly important for physicians treating unknown cases of pesticide poisoning.

## 5 Conclusion

Imidacloprid is the most widely used neonicotinoid insecticide (Abd-Elhakim et al., 2023). Although it is well known for its safety, there is growing evidence of its toxicity. An appropriate management for imidacloprid poisoning has not yet been established, and more clinical evidence is needed to prove its safety and toxicity. Toxicant detection is of great value for the diagnosis, assessment of disease severity, and treatment effects. Hemoperfusion, glucocorticoids, and other comprehensive treatments are effective against imidacloprid poisoning.

## Data Availability

The original contributions presented in the study are included in the article/Supplementary Material, further inquiries can be directed to the corresponding author.
