# Supplementary figures and images for: Treatment of an accident of imidacloprid poisoning
Source: Front Pharmacol. 2024 Jul 24;15:1421437. doi: 10.3389/fphar.2024.1421437 (PMC11303190; doi:10.3389/fphar.2024.1421437)

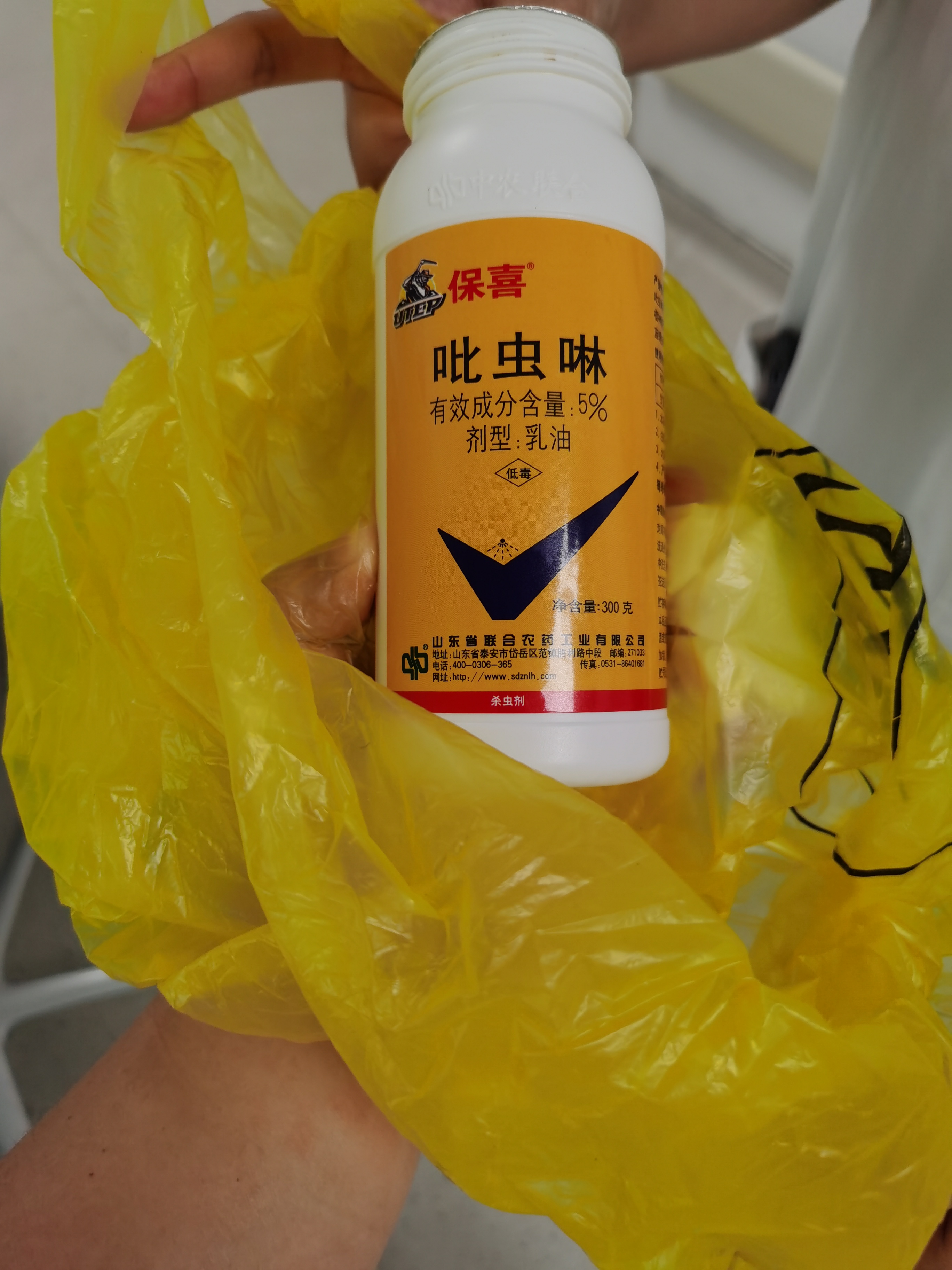

Supplement: Supplementary file 1 [file Image1.JPEG]
